# Supplementary material for: Urea as a Cocrystal Former—Study of 3 Urea Based Pharmaceutical Cocrystals
Source: Pharmaceutics. 2021 May 7;13(5):671. doi: 10.3390/pharmaceutics13050671 (PMC8151602; doi:10.3390/pharmaceutics13050671)
Supplement: Supplementary file 1 [file pharmaceutics-13-00671-s001.zip › pharmaceutics-1201408-supp.pdf]

pharmaceutics

MDPI

Supplementary Materials: Urea As a Cocrystal Former—Study of 3 Urea Based Pharmaceutical Cocrystals

Fucheng Leng, Koen Robeyns and Tom Leyssens

CCDC 2076912-2076914 contain the supplementary crystallographic data for this paper. These data can be obtained free of charge from The Cambridge Crystallographic Data Centre via www.ccdc.cam.ac.uk/structures

Table S1. List of APIs used in cocrystal screen.

Naproxen	Ketoprofen	Ibuprofen
Aspirin	Caffeine	Phenacetine
Acetaminophen	Theophylline(reported)	Levetiracetam
Pramiracetam	Fasoracetam	Carphdon
Sunifiram	Aniracetam	Piracetam
Nefiracetam	Coluracetam	Nicotinamide(reported)
Idebenone	Fladrafinil	Oxcarbamazepine
Salicylamide	L-penicillamine	Diprophylline
Primidone	Xanthine	Sulfathiazole
Theophylline-7-acetic acid	Hydroquinone(reported)	Phloroglucinol
Isonicotinic acid	Etofylline	Dyphylline
2-Ehoxybenzamide	3-Hydroxy-2-naphthoic acid(new)	Sulfacetamide
L-Ascorbic acid	4-Nitroaniline	Anthranilic acid
Acetanilide	Formanilide	Thiobenzamide
Nicotinic acid	3-Aminobenzamide	Praziquantel
S-methyl-L-cysteine	L-Pyroglutamic acid	Indoprofen
Lidocaine	Ellagic acid(new)	Flurbiprofen
Quercetin	Rutin	Catechin(new)
Epicatechin	Salicylic acid(reported)	

Table S2. Crystallographic data of UE obtained from Rietveld refinement

Chemical formula	$C_{14}H_6O_8$, 2(CH_4N_2O)
Formula mass	315.32
Crystal system	Triclinic
a/Å	11.6649(15)
b/ Å	6.8628(4)
c/ Å	6.1953(4)
α/°	72.912(4)
β/°	114.354(4)
γ/°	115.491(3)
Unit cell volume/A3	403.74(6)
Temperature/K	293
Space group	*P* -1
Z (formula units per unit cell)	1
Radiation type	Cu Kα1
2θ range/°	2.09–62.285
Number of reflections	557
R factors not corrected for background	
R /%	5.0768
wR /%	7.1968
Rexp /%	4.0738
χ^2	3.1209
R factors background corrected	
Rp /%	20.7037
Rwp /%	19.1944
Re /%	10.8651
χ^2	3.2511

Pharmaceutics **2021**, *13*, 671; https://doi.org/10.3390/pharmaceutics13050671 www.mdpi.com/journal/pharmaceutics

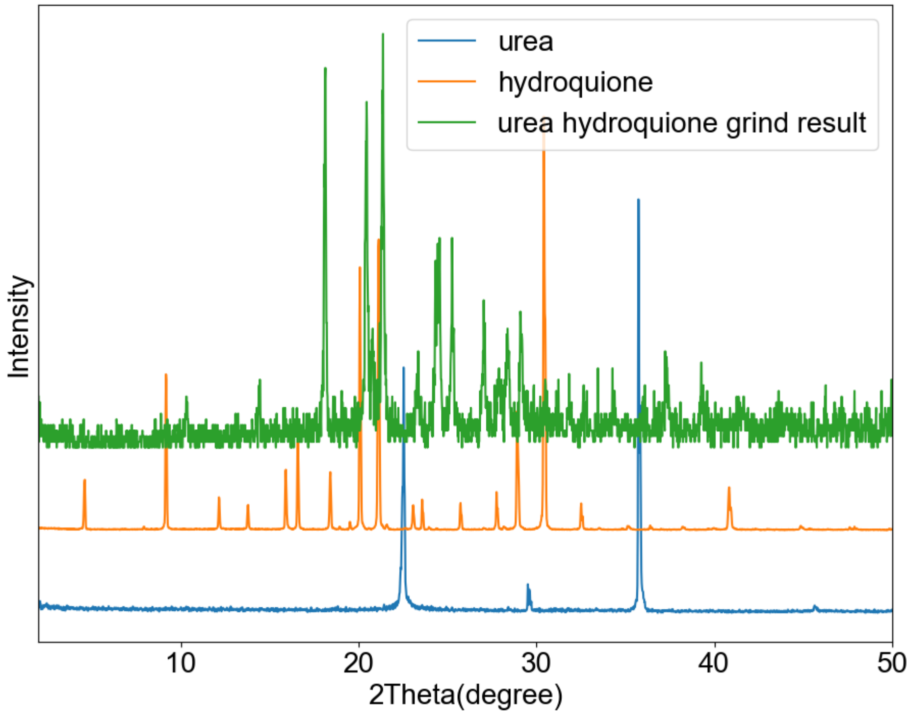

Figure S1. PXRD profiles of urea, hydroquion, and urea hydroquione cocrystal obtained by grinding.

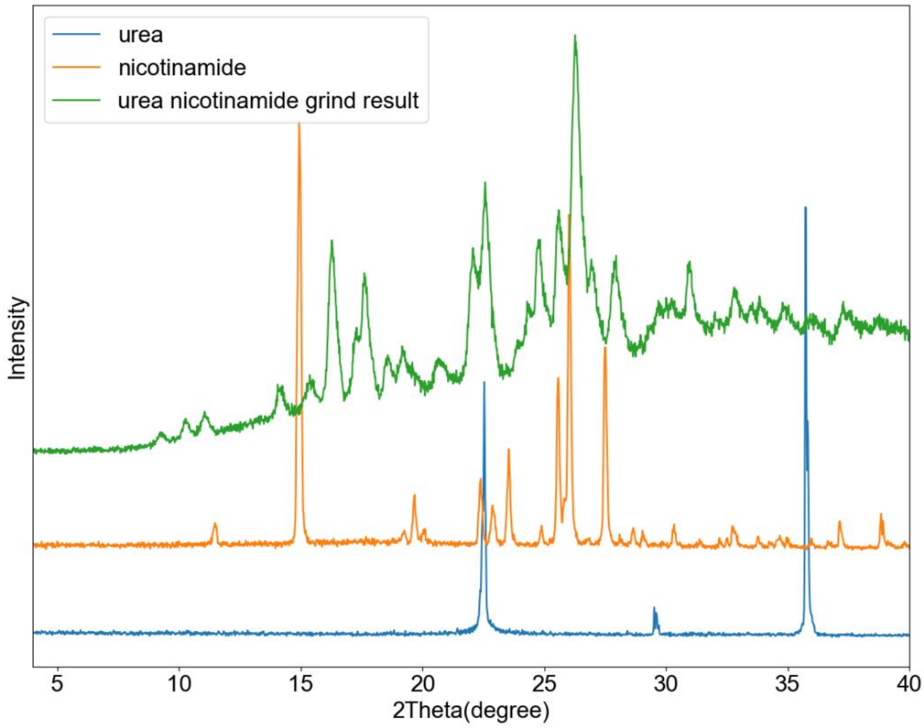

Figure S2. PXRD profiles of urea, nicotinamide, and urea nicotinamide cocrystal obtained by grinding.

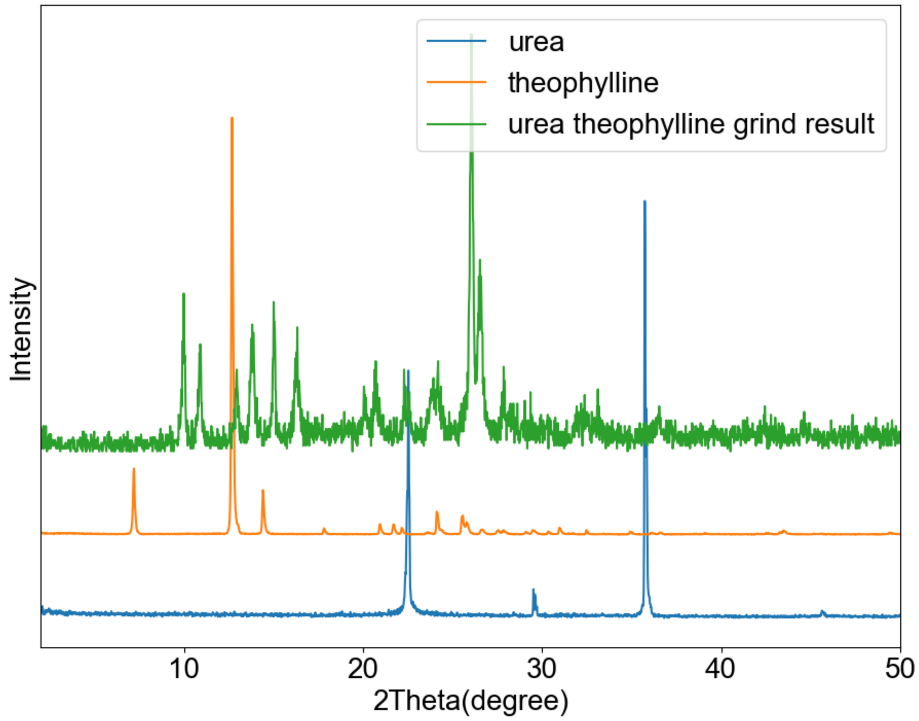

Figure S3. PXRD profiles of urea, theophylline, and urea theophylline cocrystal obtained by grinding.

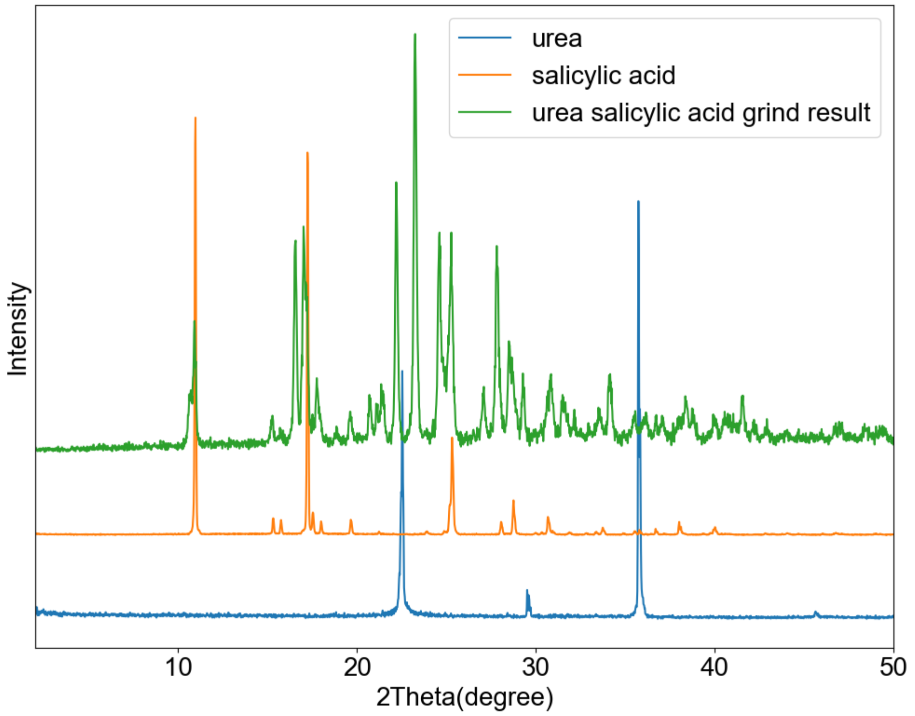

Figure S4. PXRD profiles of urea, salicylic acid, and urea salicylic acid cocrystal obtained by grinding.

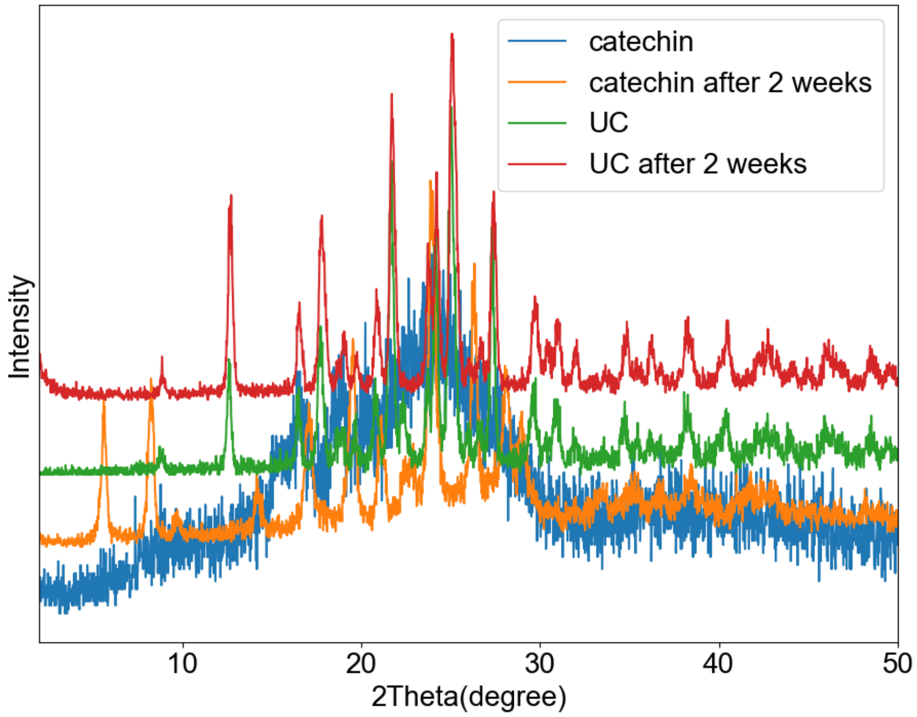

Figure S5. PXRD profiles of catechin and UC before and after storage at 25 °C and 75% relative humidity for two weeks.

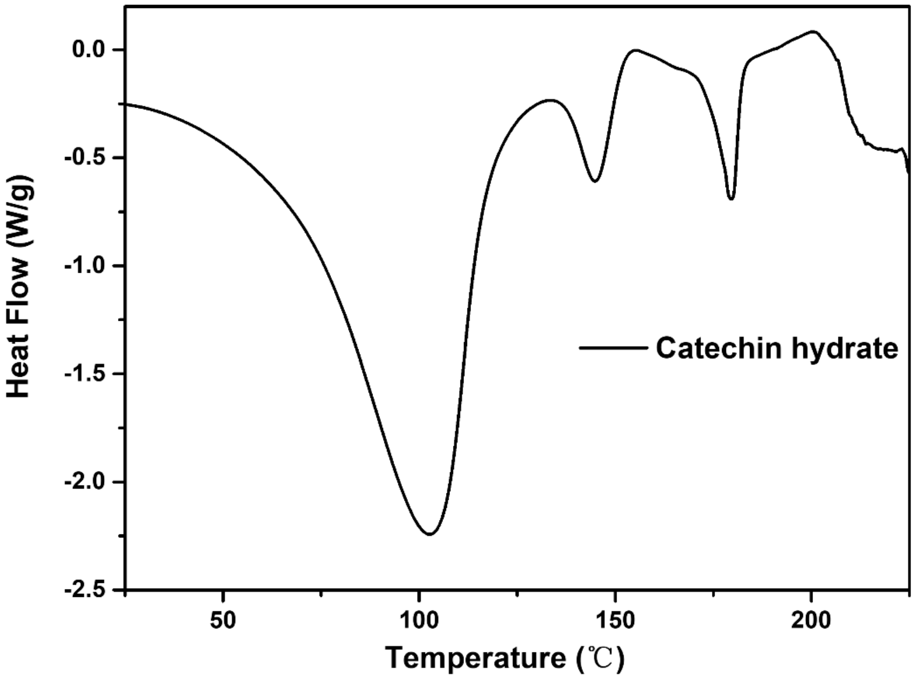

Figure S6. DSC thermogram of catechin hydrate.

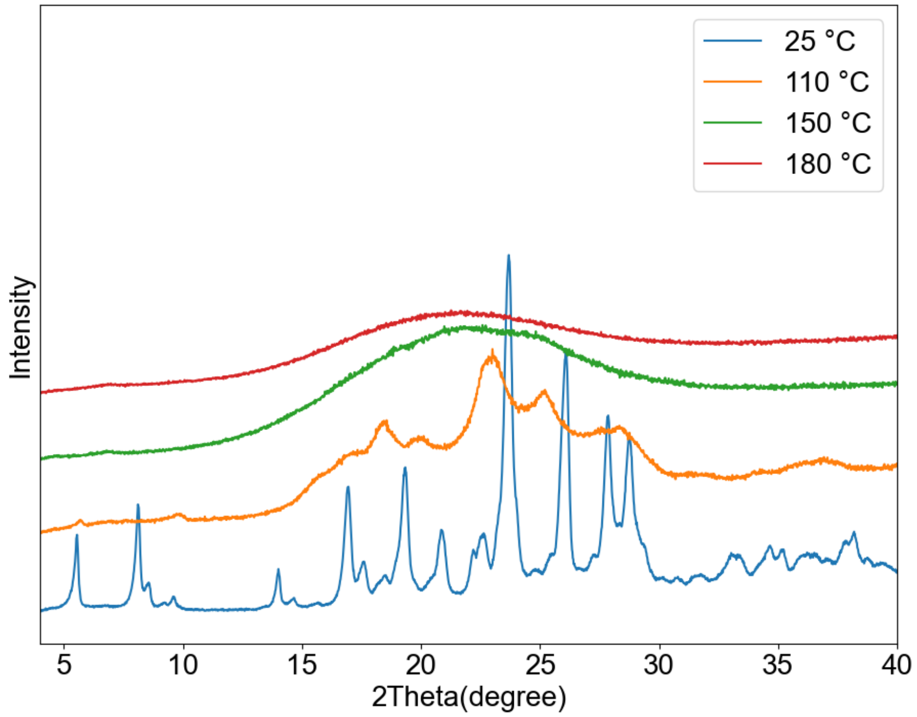

Figure S7. VT-PXRD profile of catechin hydrate.

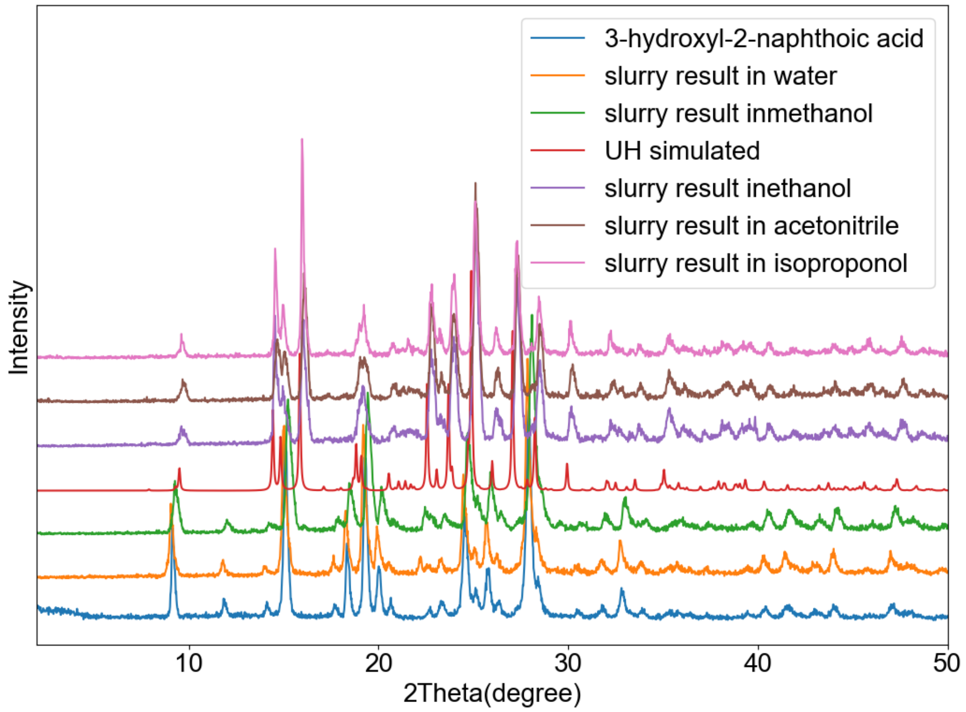

Figure S8. PXRD of UH congruency experiments in different solvents.

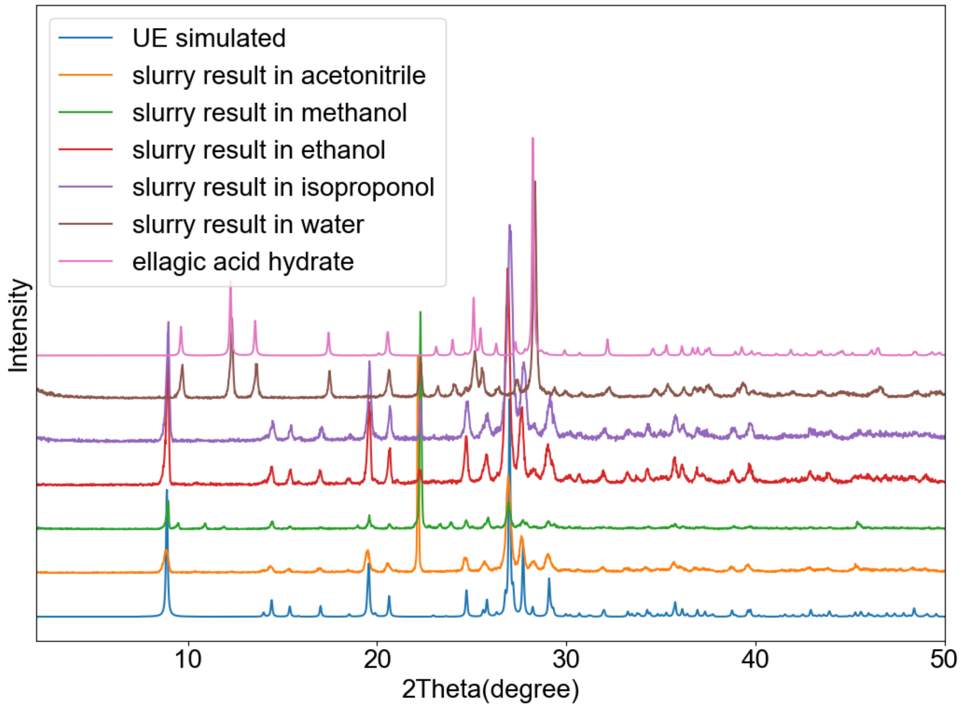

Figure S9. PXRD of UE congruency experiments in different solvents.

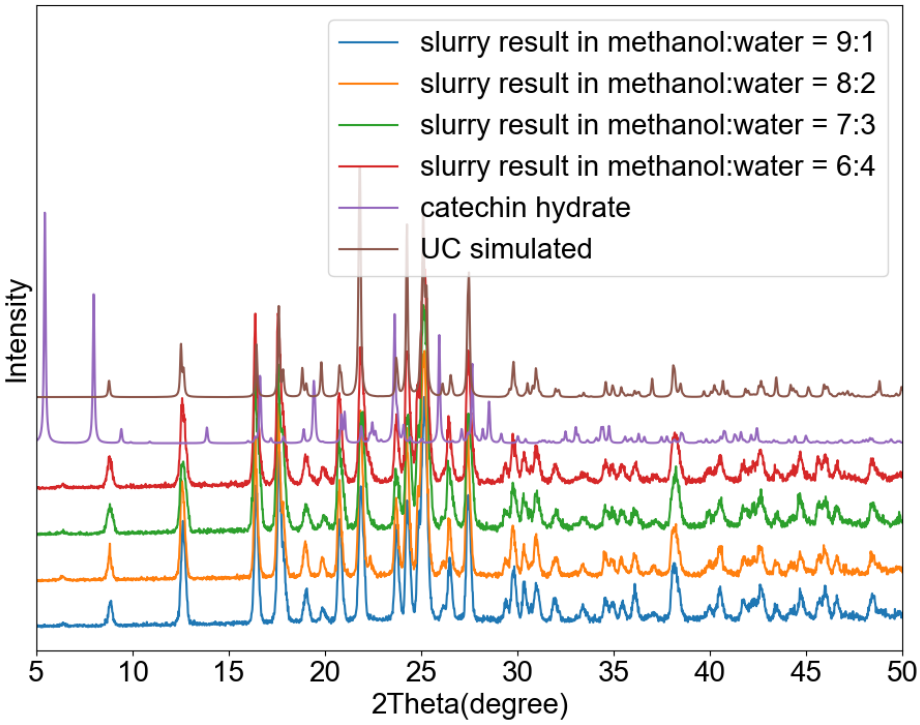

Figure S10. PXRD of congruency experiments of UC in water/methanol mixed solvents (from 1:9 to 4:6).

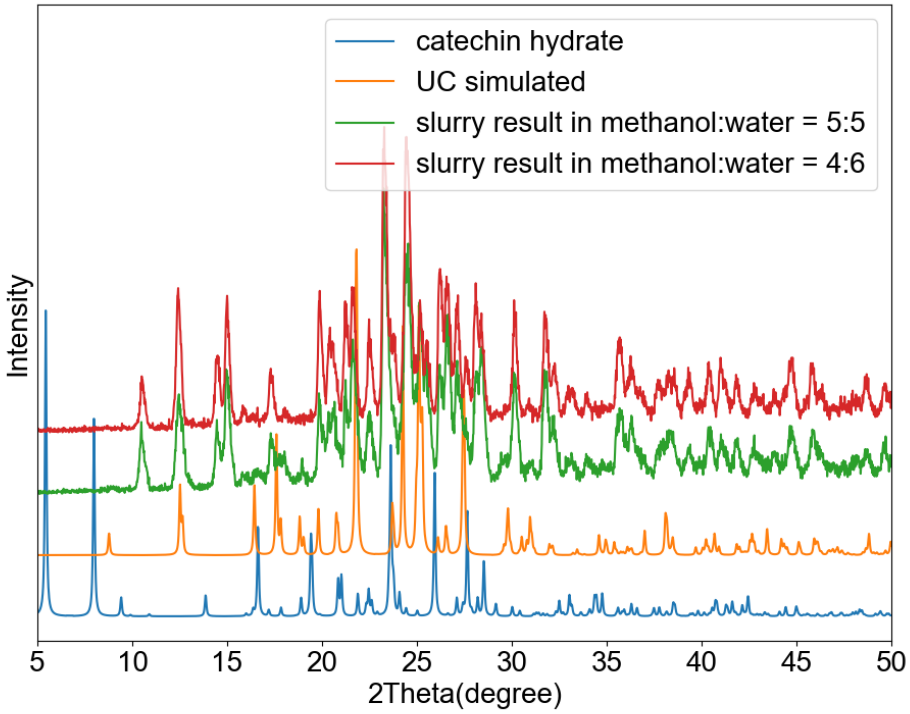

Figure S11. PXRD of congruency experiments of UC in water/methanol mixed solvent (from 5:5 to 6:4).

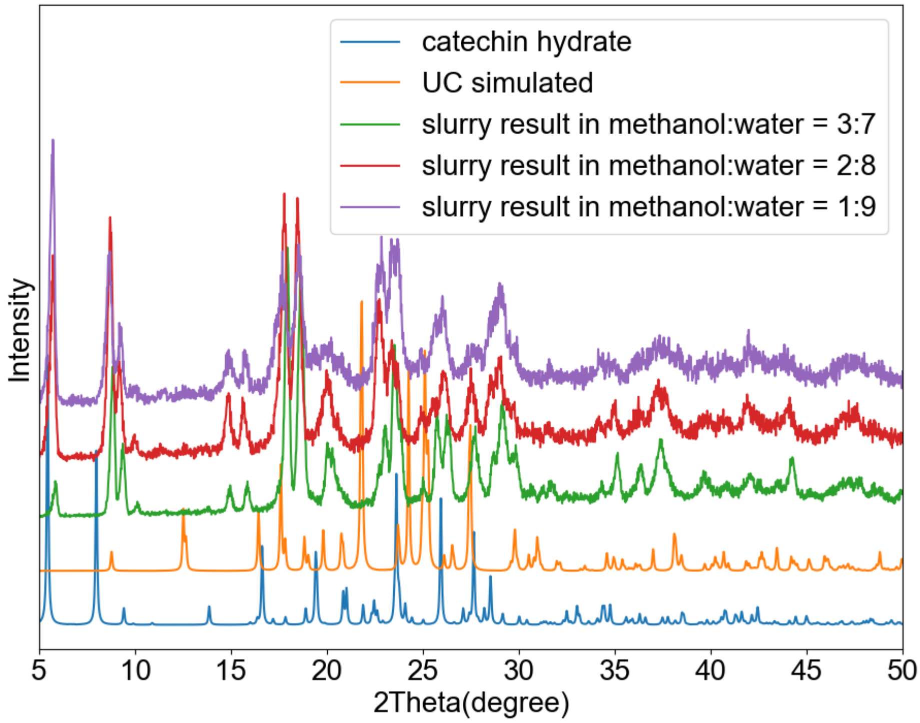

Figure S12. PXRD of congruency experiments of UC in water/methanol mixed solvent (from 7:3 to 9:1).

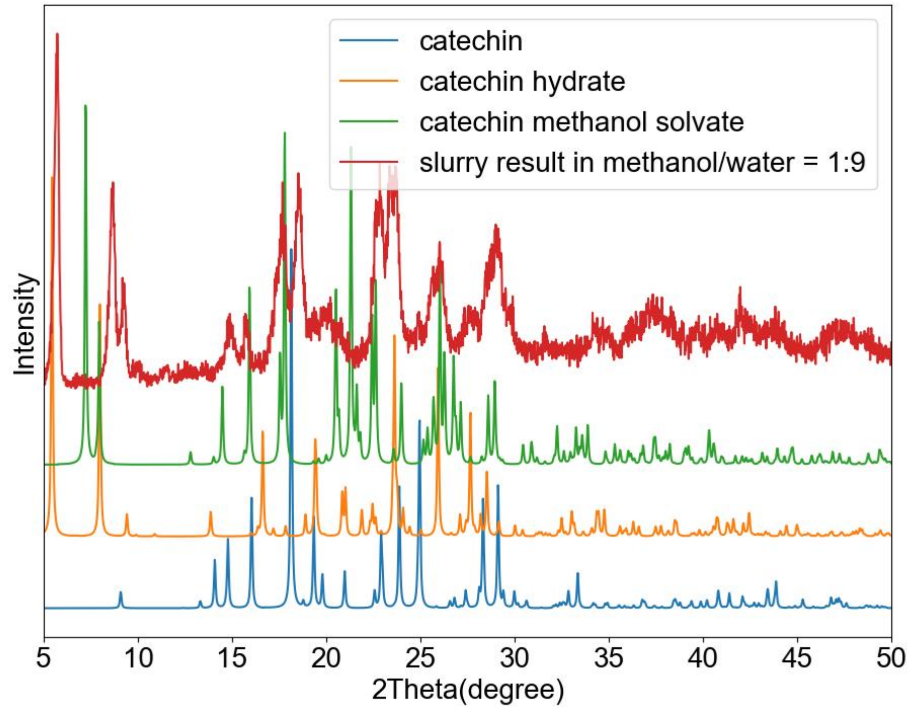

Figure S13. PXRD profiles of the catechin solvate obtained from slurrying urea and catechin in mixed solvents of methanol and water (1:9), and reported catechin polymorph or solvate.

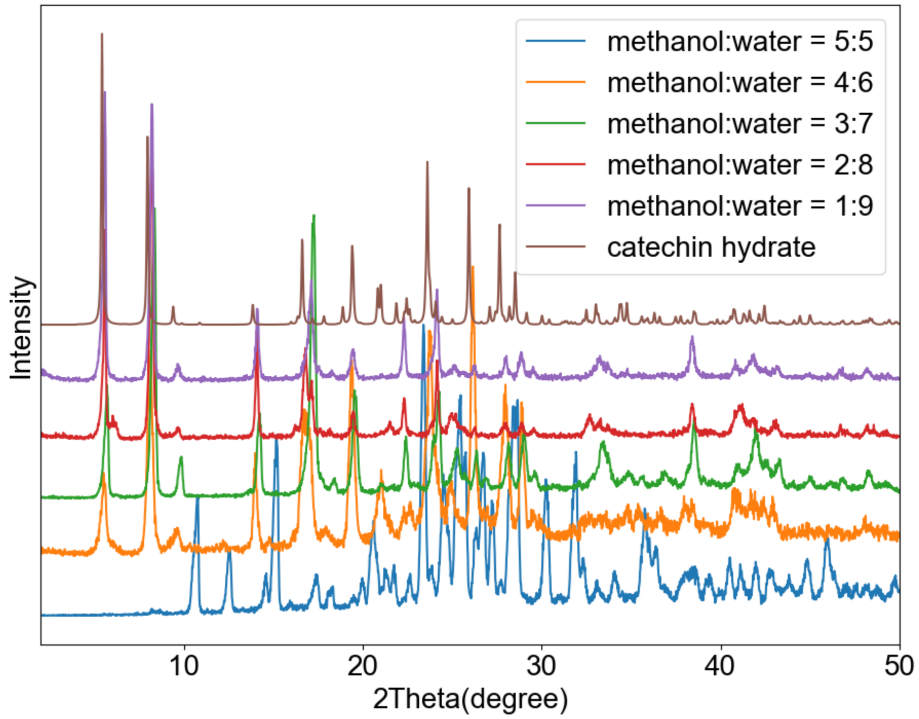

Figure S14. PXRD profiles of catechin slurry results in methanol/water mixed solvents.

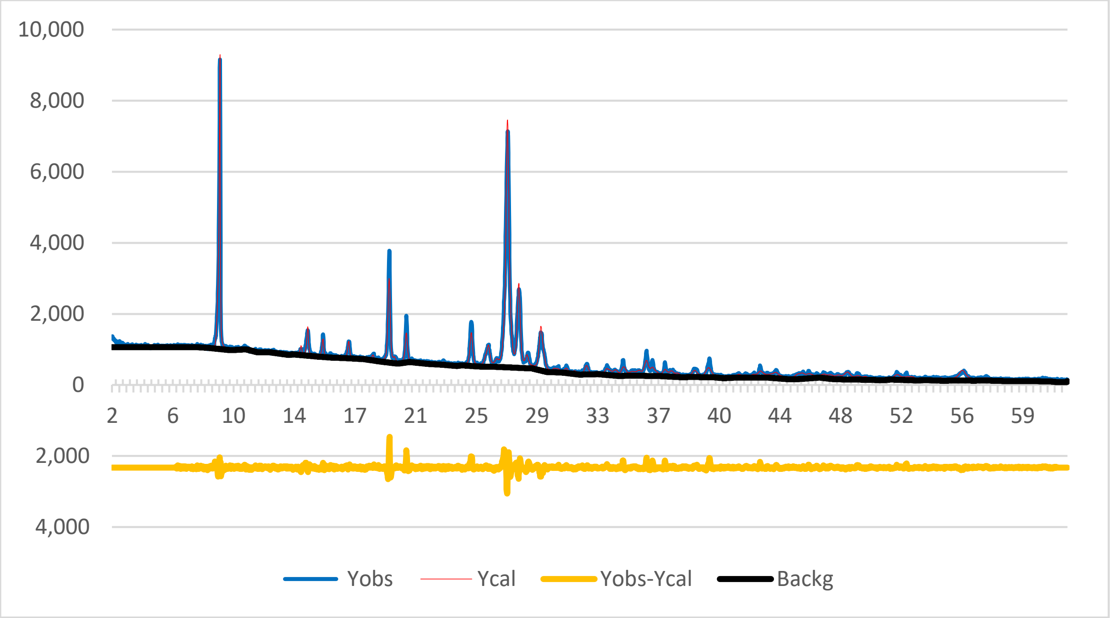

Figure S15. XRPD structure resolution data for the ellagic acid urea cocrystal.
